# Reply to Siqueira-Batista and Gómez: Underscoring the importance of biosafety for the return of Martian samples

**DOI:** 10.1073/pnas.2508447122

**Published:** 2025-05-27

**Authors:** Charles W. Whetsel, Joel S. Levine, Stephen J. Hoffman, Erik L. Antonsen

**Affiliations:** ^a^Planetary Sciences Directorate, Jet Propulsion Laboratory-California Institute of Technology, Pasadena, CA 91011; ^b^Department of Applied Sciences, William and Mary, Williamsburg, VA 23187; ^c^Engineering and Technology Group, The Aerospace Corporation, Houston, TX 77058; ^d^Division of SPEAR Medicine, Department of Emergency Medicine, Massachusetts General Hospital, Boston, MA 02114

The authors strongly endorse the protection of the Earth’s biosphere against harmful biological contamination by potential extraterrestrial life or bioactive molecules using the principle of assured containment for return of any unsterilized Martian materials until the prospects of future samples containing unidentified microorganisms are better understood, a point underscored by Siqueira-Batista, et al. (1). While not discussed in detail in our recent paper (2), the literature shows that such a principle has been and remains central to the planning by NASA and ESA for the robotic return of Martian samples, such as those collected to date by the Mars 2020 Perseverance rover mission (3–8), as well as in the conduct of scientific investigations following the return of such samples and in the protocols for how such samples should be handled and characterized related to biosafety concerns and their implications for future missions to Mars (3–5, 9, 10).
